# Retraction: Evaluation of the Replication, Pathogenicity, and Immunogenicity of Avian Paramyxovirus (APMV) Serotypes 2, 3, 4, 5, 7, and 9 in Rhesus Macaques

**DOI:** 10.1371/journal.pone.0244045

**Published:** 2020-12-14

**Authors:** The PLOS ONE Editors

After this article [1] was published, concerns were raised about results reported in Fig 1.

Specifically:

- The following lanes appear similar:
  ○ Day 0 lanes for Animals #2, 3 in the rAPMV-4 panels
  ○ Day 0 data reported for Animal #1, rAPMV-4, appear to match the image data for Day 0, Animal #4, rAPMV-4/Fc-BC. This issue was confirmed in the University of Maryland’s investigation of this work.
  ○ Day 0 lanes for Animals #1, 2, 3, 4 in WtAPMV-7 panel and Animals #1, 2, 3, 4 in the rAPMV-7/Fcs-5B panel
  ○ Day 28 data for Animals #1 and #3 in the WtAPMV-7 panel
- There appear to be vertical discontinuities suggestive of image splicing between lanes in all panels of Fig 1.
- Concerns were raised about controls and aspects of the experimental design for the Fig 1 results. No loading control data or pre-immune/no antibody control blots were reported to support the western blot results in Fig 1. Furthermore, the authors explained that virus samples for each of the APMV strains were run together on the same blot; the membranes were cut into individual strips and incubated individually with Day 0 or Day 28 sera from each monkey; after blot development by chemiluminescence, figures were generated by compiling data obtained on multiple blot strips. This image splicing was not declared in the figure legend, and the validity of comparisons between Day 0 and Day 28 results is in question since results were obtained using different blot strips and no loading control data were reported.

The authors commented that Day 0 data looked similar across experiments and that the same control data were reused to represent different results due to errors in figure assembly. Regarding the Day 28 results (Animals #1 and #3 in the WtAPMV-7), the authors stated that the original results for these animals appear similar but they stand by these results as reported in the figure.

The University of Maryland investigated this work and confirmed that several lanes are duplicated in this figure, including control (Day 0) lanes in several panels and the two Day 28 lanes in the WtAPMV-7 panel.

Raw image data were provided in support of Fig 1. The image data supported the Day 28 results reported in most panels of the figure. The Day 0 data did not match the results in the figure in most cases, and for some control lanes we were unable to confirm verifiable image content. Overall, the issues about experimental controls and Day 28 results for WtAPMV-7 were not resolved, and we remain concerned about the extent of image data reuse in the published figure.

In light of the above concerns, the *PLOS ONE* Editors retract this article.

UJB agreed with retraction. SKK, SKS, and PLC did not agree with retraction and stand by the article’s findings. PLC apologizes for the issues with the article. The other authors either could not be reached or did not respond directly.
